# Knowledge of Undergraduate Students About Chronic Obstructive Pulmonary Disease in King Saud bin Abdulaziz University for Health Sciences, Riyadh, Saudi Arabia

**DOI:** 10.7759/cureus.60601

**Published:** 2024-05-19

**Authors:** Nawaf Yousef Othman Aloraini, Hadi M Shaabi, Bader H Alotaibi, Mahdy W Altabyanawy, Bassam A Aldakhil, Aamir Omair

**Affiliations:** 1 College of Medicine, King Saud bin Abdulaziz University for Health Sciences, Riyadh, SAU; 2 Research, King Abdulaziz Medical City, Riyadh, SAU; 3 Medical Education/Research, King Saud bin Abdulaziz University for Health Sciences, King Abdullah International Medical Research Center, Ministry of National Guard Health Affairs, Riyadh, SAU

**Keywords:** undergraduates, questionnaire, students, knowledge, copd

## Abstract

Aims

Chronic obstructive pulmonary disease (COPD) is one of the most common causes of death worldwide. This study assesses the level of knowledge about COPD among undergraduate students that makes it different from other respiratory illnesses.

Methods

A cross-sectional study was conducted among undergraduate students at King Saud bin Abdulaziz University for Health Sciences (KSAU-HS). The Bristol Chronic obstructive pulmonary disease Knowledge Questionnaire (BCKQ) was used to evaluate the knowledge about COPD, epidemiology, symptoms, exercise, smoking, and breathlessness domains. The questionnaire was distributed among the different male colleges.

Results

There were 304 respondents from five colleges. The overall BCKQ mean score was 15.16±4.52 (maximum 30). The mean score was highest for the Colleges of Pharmacy (18.89±2.17) and Medicine (18.00±3.84), and the College of Science and Health Professions had the lowest score (11.56±5.58). The highest overall means for the different domains (max=5) were for smoking (2.19±1.2), and epidemiology (2.83±1.27), while symptoms of COPD (2.23±1.06) and breathlessness (1.96±1.13) were the lowest among the domains.

Conclusions

There was a low level of understanding among undergraduate students in general, but the Colleges of Medicine and Pharmacy had better knowledge. On the other hand, the College of Science and Health Professions had a lower score. This indicates some areas for improvement in the education program. Appropriate development in the education program is recommended, such as increasing the awareness of symptoms of COPD and other aspects of the disease.

## Introduction

Chronic Obstructive Pulmonary Disease (COPD), is a respiratory disease affecting the airways, making it harder to deliver oxygen to the body. This oxygen deprivation has been shown to heavily cause everything ranging from disruptions to sleep patterns to reduced physical ability [1]. COPD affects about 10% of women and 16% of men, accounting for almost 6% of worldwide deaths [1-2]. This makes it the third leading cause of death worldwide [2].

The major cause of COPD is smoking cigarettes. In a large study conducted in the US with a data size of 428,378, it was seen that current smokers (15.2%) were twice as likely to have COPD than former smokers (7.6%), and more than five times more likely than non-smokers (2.8%) [3]. A Chinese study showed that inhaling tobacco smoke could lead to inflammation of the airways causing significant scarification [4]. This is also true for electronic cigarettes and second-hand smoke inhalation.

According to a survey conducted in Turkey on 8342 people, approximately 49.6% of the respondents knew COPD was a respiratory disease [5], which is understandable considering the prevalence of COPD in Turkey was about 6.9% [6]. A study in South Korea of 289 people in smoking cessation clinics with mostly 94.5% men showed that the percentage of people who knew that COPD was a lung disease was 21.8% [7], even though the prevalence of the disease in Turkey is 13.2% [8].

In Turkey, out of the 8342 subjects mentioned above, only 25.2% knew that it could be treated [5]. It has been shown that people are more willing to stop smoking after learning about COPD and the effects it can have. After being informed about COPD, 84.1% of patients of the South Korean smoking-cessation clinics were more willing to stop smoking [7]. From what is available regarding the variability of COPD prevalence and public awareness, it would be expected that statistics from Saudi Arabia would differ from those of Turkey and Korea. A survey published in 2014 showed the prevalence in Saudi Arabia was 2.1% [9], which, according to the World Bank is about 742,000 people [10].

After a thorough literature review using PubMed Central and Google Scholar’s unified search engine, searching for keywords “COPD awareness” and “COPD knowledge”, it was found that there was a gap in the information available in regard to this aspect of the disease. Not enough published article assessing the public awareness of COPD in Saudi Arabia was found, which should have a sufficient number of studies because it is the 3rd cause of death in the world according to WHO, and it can be preventable by early recognition and education [11-12].^​^ This study aimed to assess the awareness of COPD among students at King Saud bin Abdulaziz University for Health Sciences (KSAU-HS). The study assessed and measured their knowledge of COPD and its risk factors.

## Materials and methods

A cross-sectional study was conducted in King Saud bin Abdulaziz University for Health Sciences, Riyadh, Saudi Arabia to assess the students’ knowledge about Chronic Obstructive Pulmonary Disease. Namely, College of Science and Health Professionals, College of Medicine, College of Pharmacy, College of Dentistry, College of Applied Medical Sciences. There are approximately 1850 students studying in the basic and pre-clinical, clinical phase during the academic year 2022-2023. The required sample size was estimated to be 319 students for a population of 1850 with a confidence interval of 95%, an expected percentage of 50% for the outcome variable, and a margin of error of 5% calculated by the Raosoft sample size calculator (Raosoft Inc., Seattle, USA) [13].

Non-probability convenience sampling was used for the study. The questionnaire was handed by the co-investigators to students in the different colleges in King Saud bin Abdulaziz University for Health Sciences in Riyadh, Saudi Arabia. The inclusion criteria were male students, Riyadh campus, second year or lower from College of Medicine, College of Science and Health Professions, College of Pharmacy, College of Dentistry, and College of Applied Medical Sciences. All those students who met the criteria and were available during data collection were included in the study.

Data were collected by using a self-administered questionnaire in the English language. The questionnaire was taken from the Bristol COPD Knowledge Questionnaire [14]. The questionnaire contains six domains each having five true/false questions designed to evaluate the students’ knowledge. The questionnaire asked about the following domains: chronic obstructive pulmonary disease (COPD), COPD disease, epidemiology, symptoms, breathlessness, exercise, and smoking. The main outcome variables included knowledge of chronic obstructive pulmonary diseases as known or unknown, and attitude as high-risk diseases or non-harmful illnesses. The grouping variables included different colleges, smoking status, and the year of studying.

The data was entered using Microsoft Excel (Microsoft Corporation, Redmond, USA) and then transferred to IBM SPSS v25 (IBM Corp., Armonk, USA) for data analysis. Categorical data (descriptive statistics) was presented as frequencies and percentages and numerical data was presented as the mean and standard deviation. The knowledge level was presented as mean ± sd for the number of correct responses for each domain and the overall total. ANOVA was used to assess the association between the outcome variables (knowledge level about COPD) and categorical data such as College. The test was considered significant if the p-value was less than 0.05.

The approval for the research was obtained from the Institutional Review Board of King Abdullah International Medical Research Centre. All the students were given the consent form along with the questionnaire. The study was completely voluntary. No compensation or benefits were given to the participants to maintain ethical standards. Information related to the personal identification of the students, including names and ID numbers, was not taken. All data collected were kept secure and only members of the research team had had any access to it.

## Results

There were 304 students who completed the survey. These included 114 (38%) from the College of Science and Health Professionals (COSHP), 101 (33%) from the College of Medicine (COM), 46 (15%) from the College of Applied Medical Sciences (CAMS), 25 (8%) from the College of Dentistry (COD), and 18 (6%) from the College of Pharmacy (COP). These are in the approximate proportion of the total number of male students in the respective Colleges as the COSHP and COM have a total of approximately 800 and 350 students as compared to around 70 and 60 respectively in the COD and COP.

**Figure 1:**
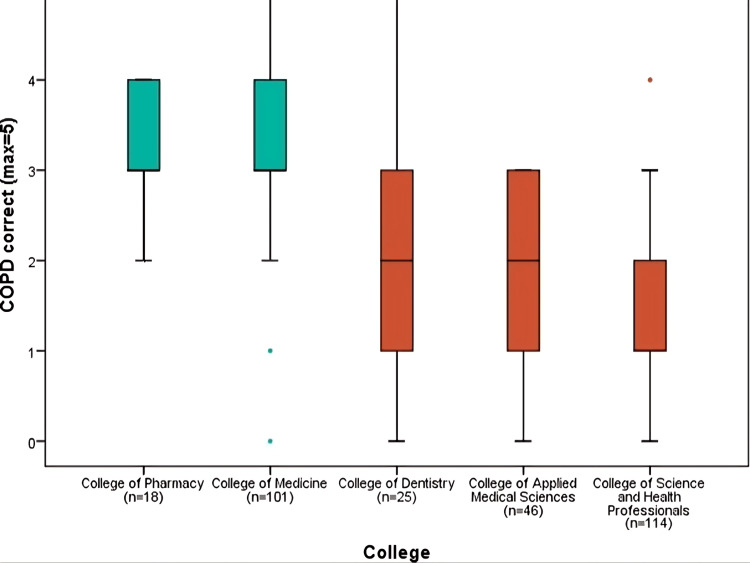
COPD domain correct responses comparison by colleges COPD: chronic obstructive pulmonary disease

Figure 1 shows the number of correct responses (max=5) for the domain (chronic obstructive pulmonary diseases), which included the differentiating features of chronic obstructive pulmonary diseases. Overall, the mean of the domain correct answer was (2.39± 1.04) (p<0.001). Two colleges had more than two answers correct, i.e., College of Medicine (3.22) followed by the College of Pharmacy (3.17) and three colleges had less than three, i.e., College of Dentistry (2.12), College of Applied Medical Sciences (1.93) and the College of Science and Health Professionals (1.53).

**Figure 2:**
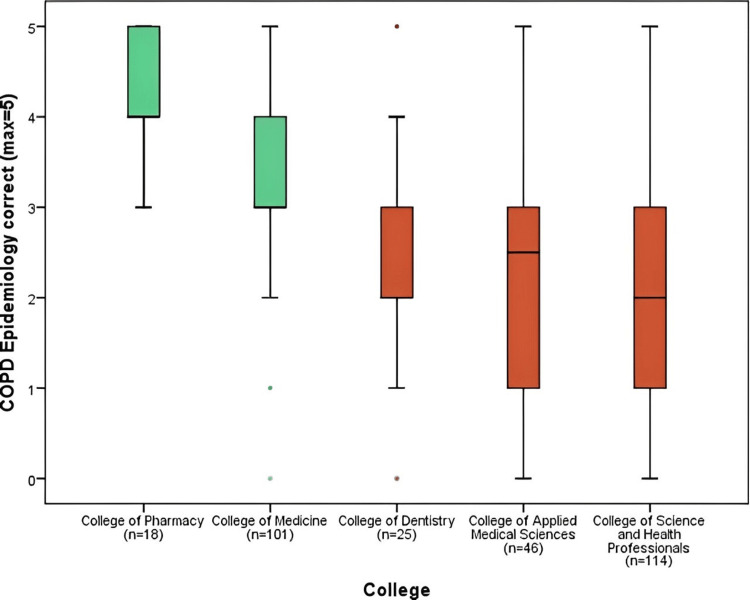
COPD epidemiology correct responses comparison by colleges COPD: chronic obstructive pulmonary disease

Figure 2 shows the number of correct responses for the domain regarding the awareness of chronic obstructive pulmonary disease's epidemiology (max=5). Three of the colleges had less than three answers correct i.e., College of Dentistry (2.40), College of Applied Medical Sciences (2.28), and College of Science and Health Professionals (2.11). The College of Pharmacy had the highest correct answer (4.11), followed by the College of Medicine (3.29) (p<0.001). The chronic obstructive pulmonary diseases epidemiology domain recorded one of the highest correct answers mean of (2.83) as compared to the rest of the domains.

**Figure 3:**
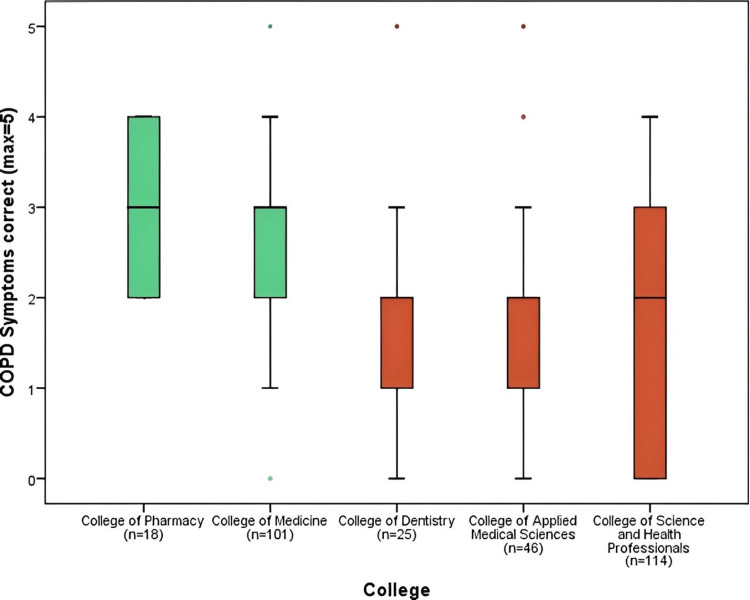
COPD symptoms correct responses comparison by colleges COPD: chronic obstructive pulmonary disease

Figure 3 presents the results of correct responses (max=5) to the symptoms of chronic obstructive pulmonary diseases. The College of Pharmacy had a mean of (3.00 ± 0.77) answers correct and the College of Medicine (2.76 ± 0.99), which were significantly higher than the mean scores for the College of Dentistry (1.84), College of Applied Medical Sciences (1.83), and College of Science and Health Professionals (1.72). Overall, the mean of domain correct answers was (2.23) which is the second lowest in comparison to the other domains. 

**Figure 4:**
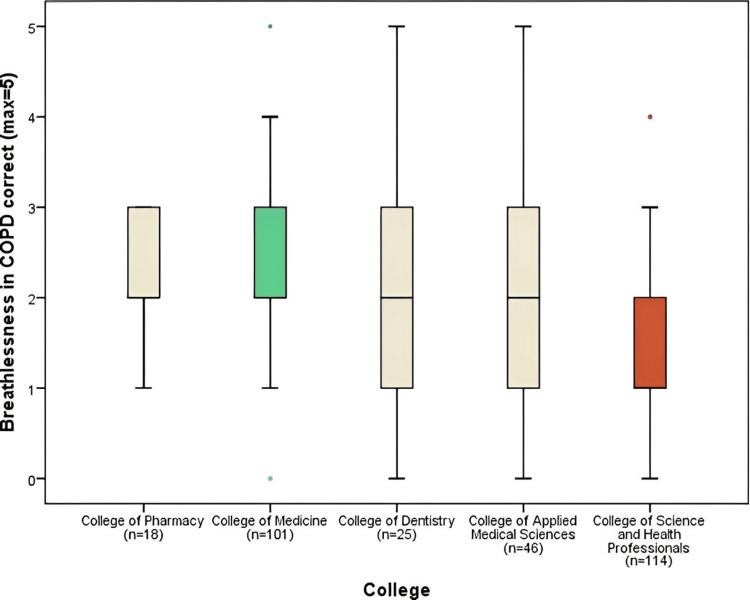
Breathlessness in COPD correct responses comparison by colleges COPD: chronic obstructive pulmonary disease

Figure 4 presents the results of correct responses (max=5) for the domain regarding the prevalence of breathlessness in chronic obstructive pulmonary disease. None of the five groups had a mean of 3 or more correct answers. The highest mean was for the College of Medicine (2.36 ± 1.18) which was significantly higher than that for the College of Science and Health Professionals (1.51 ± 1.15) (p<0.001) Overall, the breathlessness in the chronic obstructive pulmonary diseases domain had a mean of (1.96), which is the lowest score in comparison to the other domains. 

**Figure 5:**
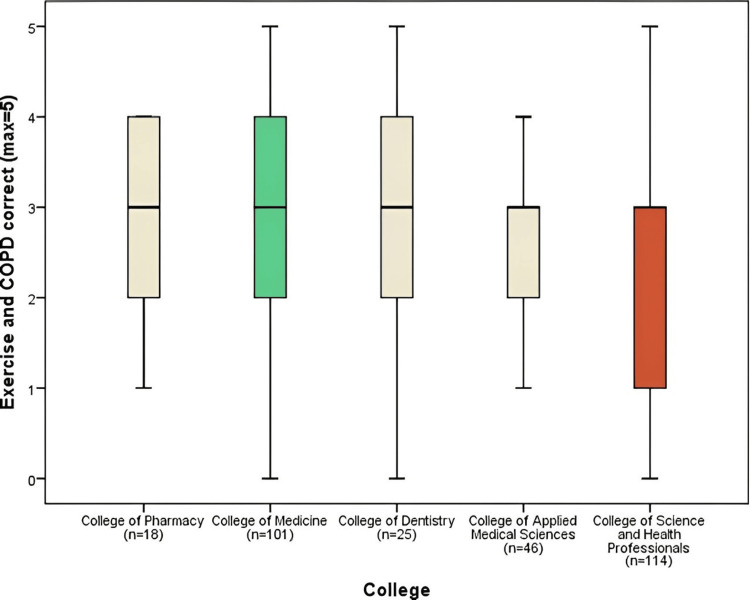
Exercise and COPD correct responses comparison by colleges COPD: chronic obstructive pulmonary disease

Figure 5 shows the results of the correct responses (max=5) for the domain of exercise and chronic obstructive pulmonary disease. The College of Medicine (3.05 ± 1.09) had an average score greater than 3, which was significantly higher than the College of Science and Health Professionals (2.34 ± 1.46) (p=0.003). The mean of all the groups in the exercise and chronic obstructive pulmonary diseases domain was (2.66). 

**Figure 6:**
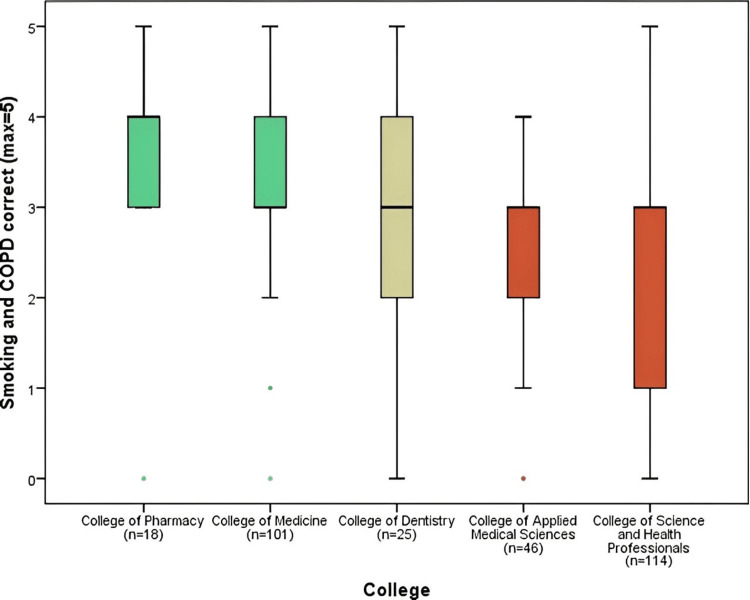
Smoking and COPD correct responses comparison by colleges COPD: chronic obstructive pulmonary disease

Figure 6 displays the results of correct responses (max=5) for the domain of smoking and chronic obstructive pulmonary diseases. The College of Pharmacy (3.56 ± 1.04) and the College of Medicine (3.33 ± 0.86) had a mean of more than three answers correct, which was significantly greater than the means for the College of Applied Medical Sciences (2.52 ± 1.41) and the College of Science and Health Professionals (2.35 ± 1.38) (p<0.001). Smoking and chronic obstructive pulmonary diseases domain had a correct answer mean of (2.91), which is the highest among all the domains. 

Table 1 shows a comparison between the five Colleges for the total correct responses (max=30) in all six domains about chronic obstructive pulmonary disease. The College of Pharmacy (18.89 ± 2.17) and the College of Medicine (18.00 ± 3.84) had significantly (p<0.001) higher correct responses as compared to the other three Colleges: the College of Dentistry (13.48 ± 5.34), College of Applied Medical Sciences (13.09 ± 5.70), and the College of Science and Health Professionals (11.56 ± 5.58).

**Table 1:** Mean total score by college

Total by colleges		Total Correct Score (Max=30)		
	n	Mean	sd	p-value
College of Pharmacy	18	18.89	2.17	<0.001
College of Medicine	101	18.00	3.84	
College of Dentistry	25	13.48	5.34	
College of Applied Medical Sciences	46	13.09	5.70	
College of Science and Health Professionals	114	11.56	5.58	

Table 2 shows that most participants answered in COPD there is usually gradual worsening over time n=212 (70%), in COPD the word “chronic” means it is severe n=200 (66%), COPD can be caused by occupational dust exposure n=209 (69%) correctly while COPD can only be confirmed by breathing test n=93 (31%) and in COPD oxygen levels in the blood are always low n=57 (19%) were not answered correctly. 

**Table 2:** COPD and COPD epidemiology correct responses (N=304). COPD: chronic obstructive pulmonary disease

COPD	CORRECT RESPONSE	n	%
In COPD there is usually gradual worsening over time	TRUE	212	70%
In COPD the word ''chronic'' means it is severe	FALSE	200	66%
COPD is unusual in people less than 40 years old	TRUE	136	45%
COPD can only be confirmed by breathing test	TRUE	93	31%
In COPD oxygen levels in the blood are always low	FALSE	57	19%
COPD Epidemiology	CORRECT RESPONSE	n	%
COPD can be caused by occupational dust exposure	TRUE	209	69%
More than 80% of COPD cases are caused by cigarette smoking	TRUE	180	59%
Longstanding asthma can develop into COPD	TRUE	153	50%
Women are less vulnerable to the effect of cigarette smoking than men	FALSE	141	46%
COPD is commonly an inherited disease	FALSE	129	42%

Table 3 shows that most participants answered fatigue, 230 (76%), and wheezing, 227 (75%) correctly while the swelling of ankles, 96 (32%), rapid weight loss 68 (22%), and crushing chest pain 38 (13%) were not answered well as less than 40% of respondents answered correctly. Most students answered breathlessness is primarily caused by a narrowing of the bronchial tube, 189 (62%) but were wrong about breathlessness, meaning that oxygen levels are low, 63 (21%), and that severe breathlessness prevents travel by air, 43 (14%) in comparison to the others. 

**Table 3:** COPD symptoms and breathlessness in COPD correct responses (N=304). COPD: chronic obstructive pulmonary disease

COPD Symptoms	CORRECT RESPONSE	n	%
Fatigue (tiredness)	TRUE	230	76%
Wheezing	TRUE	227	75%
Swelling of ankles	FALSE	96	32%
Rapid weight loss	FALSE	68	22%
Crushing chest pain	FALSE	38	13%
Breathlessness in COPD	CORRECT RESPONSE	n	%
Breathlessness is primarily caused by a narrowing of the bronchial tubes	TRUE	189	62%
Breathlessness is a normal response to exercise	TRUE	147	48%
Breathlessness can be worsened by eating large meals	TRUE	142	47%
Breathlessness means that oxygen levels are low	FALSE	65	21%
Severe breathlessness prevents travel by air	FALSE	43	14%

Table 4 shows that most participants correctly answered exercise helps relieve depression i.e. 249 (82%) and exercise can help maintain bone density, 188 (62%) while exercise should be stopped if it makes one breathless, 50 (16%) were mostly answered wrong. Most students answered stopping smoking will slow down further lung damage, 252 (83%), stopping smoking will reduce the risk of heart disease 251 (83%), and Stopping smoking is pointless as the damage is done 221 (73%) correctly while Nicotine replacement therapy is the only available prescription 110 (36%) and Stopping smoking usually results in improved lung function 20 (7%) wasn’t well answered.

**Table 4:** Exercise and COPD, and Smoking and COPD correct responses (N=304). COPD: chronic obstructive pulmonary disease

Exercise and COPD	CORRECT RESPONSE	n	%
Exercise helps relieve depression	TRUE	249	82%
Exercise can help maintain your bone density	TRUE	188	62%
Exercise should be avoided as it strains the lungs	FALSE	160	53%
Walking is better exercise than breathing exercises to improve fitness	TRUE	159	52%
Exercise should be stopped if it makes you breathless	FALSE	50	16%
Smoking and COPD	CORRECT RESPONSE	n	%
Stopping smoking will slow down further lung damage	TRUE	252	83%
Stopping smoking will reduce the risk of heart disease	TRUE	251	83%
Stopping smoking is pointless as the damage is done	FALSE	221	73%
Nicotine replacement therapy is the only available prescription	FALSE	110	36%
Stopping smoking usually results in improved lung function	FALSE	20	7%

## Discussion

The aim of the study was to assess the general understanding of undergraduate students about Chronic Obstructive Pulmonary Disease (COPD) at King Saud bin Abdulaziz University for Health Sciences, Riyadh, Saudi Arabia. We found that undergraduate students had poor awareness of COPD. The total mean of students was less than 15 out of 30, which is low compared to other studies. the results of this study were influenced by the student's College and the time of completing the questionnaire. The Bristol COPD knowledge questionnaire was approved by 18 out of 24 health professionals and was thought to provide a satisfactory test of knowledge in a related study assessing the consistency and validity of the questionnaire [14].

As mentioned above, different studies in different settings around the world show similar results, which is a poor understanding of the disease. Previous studies found similar results in many different departments. To be specific, a study was done on final-year medical students in Spain, and their understanding of chronic obstructive pulmonary diseases. The study results indicate a moderate level of understanding as opposed to the poor knowledge level in our population [15]. Another study was done on nurses in different hospitals in Greece using the same questionnaire, and although their understanding of the disease is an important aspect as they are a crucial part in educating patients on COPD, their level of understanding was found to be poor, and the Pearson correlation coefficient for the test-retest suggests that repetition on a larger population will yield similar results [16]. To confirm, a similar study was done on internal medicine nurses in a tertiary hospital in China. The results yielded a deficit in COPD-related knowledge that needs to be strengthened [17]. There was a study on the awareness of COPD and its risk factors among the adult population of the Aseer Region, Saudi Arabia. Almost less than one-third of the population had ever heard of the disease, and awareness regarding it as a whole was low, but keep in mind they weren’t medical students; however, their level of awareness needs an urgent improvement [18].

It was discovered that the students had more knowledge about smoking than other domains. A similar study conducted by Al-Naggar and Kadir (2013) showed that the percentage of teachers' awareness of smoking was high [19]. And where the College of Pharmacy and Medicine obtained the highest percentages, this may be due to the nature of the specialization and the curriculum that focuses on the pathology of diseases, and the College of Dentistry, the College of Applied Medical Sciences, and the College of Sciences and Health Professions have the lowest percentage for their level of knowledge about smoking and COPD. The results for most domains were similar to the smoking domain on epidemiology, exercise, and COPD symptoms, but the difference was in differentiating features known as in the questionnaire COPD, and breathlessness domains. The only difference between them was the College of Medicine had a higher score than the College of Pharmacy.

To correctly interpret the results, some methodological considerations must be kept in mind. Although the data was collected in person to avoid selection bias, participation in the survey was voluntary, which may imply a selection bias. Also, some other points need to be considered, firstly the College of Applied Medicine has seven branches, some of which do not need to study COPD, such as occupational therapy. Another matter worth mentioning is the curriculum of our university, which may impact our results. Finally, for future studies, it will be better to do it in different locations and times or to do it only on females, unlike our study, then compare the results to improve the teaching system.

## Conclusions

The level of awareness among the respondents regarding COPD was poor, as only two colleges out of five answered more than half the questionnaire correctly, and the rest answered less than half of the questionnaire correctly. Areas of improvement are the following: breathlessness, symptoms, and differentiating the features of the disease. The results of the study call for an improvement in the students’ understanding of COPD and its risks.
